# A pharmacological rat model of recurrent pelvic pain exhibiting hyperalgesia and depression-like behaviors

**DOI:** 10.1016/j.isci.2026.115059

**Published:** 2026-03-05

**Authors:** Xiaotian Yang, Yajie Qin, Qi Zhao, Huiijin Zhao, Yinyin Ding, Bei Liu, Huifang Zhou

**Affiliations:** 1 Nanjing University of Chinese Medicine, Nanjing, China; 2 Affiliated Hospital of Nanjing University of Chinese Medicine, Nanjing, China; 3 Changchun University of Chinese Medicine, Changchun, China

**Keywords:** neuroscience, behavioral neuroscience, molecular biology, omics

## Abstract

Primary dysmenorrhea (PDM) involves recurrent pelvic pain (RPP), alongside menstruation and psychological comorbidity, yet existing models inadequately capture its recurrent nature. In this study, we established a pharmacologically induced rat model of RPP, using estradiol benzoate and oxytocin over six 4-day cycles. The RPP model produced robust and sustained writhing responses, with writhing latency dropping from 30 to 4 min (*p* < 0.001) and scores rising to 88.30 (*p* = 0.002), alongside persistent hyperalgesia (reduced mechanical and thermal thresholds, *p* < 0.05). Depression-like behaviors were observed as longer immobility time (*p* = 0.032) and decreased sucrose preference (*p* = 0.012). Reduced serotonin with brain-derived neurotrophic factor (BDNF) overexpression was identified in the dorsal root ganglia and serum, along with metabolomic dysregulation of amino acid and arachidonic acid pathways. While not replicating full human PDM pathophysiology, this model captures core features of RPP and affective comorbidity, providing a translational platform for mechanistic and therapeutic research.

## Highlights

• Pharmacological rat model shows recurrent pelvic pain with hyperalgesia and depression
• Peripheral BDNF and 5-HT dysregulation may underlie pain-depression comorbidity
• Serum metabolomics reveal altered amino acid and lipid metabolism in chronic pain
• This model provides a translational platform for chronic pelvic pain research

## Graphical abstract

**Figure:**
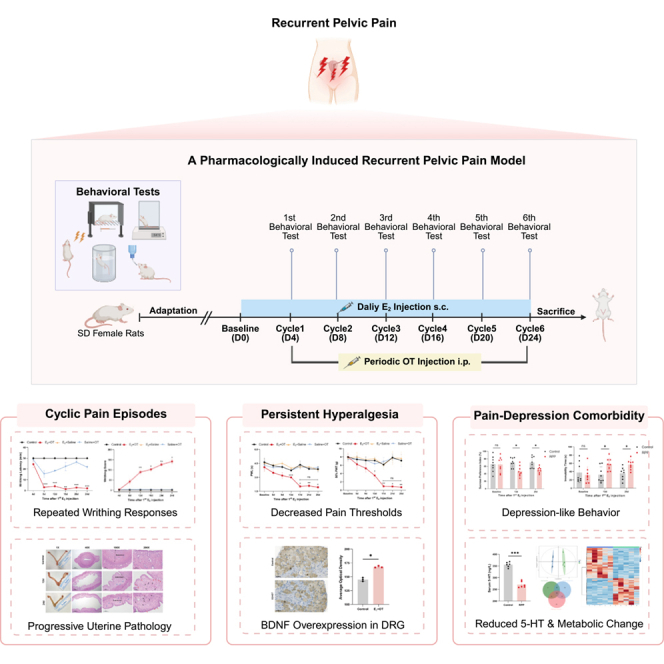

## Introduction

Primary dysmenorrhea (PDM) is characterized by recurrent pelvic pain (RPP), alongside menstruation in the absence of identifiable pelvic pathology.^1^ Affecting 24%–92% of women worldwide,^2^ PDM is often undertreated and underestimated, which significantly diminishes women’s quality of life (QoL), leading to school or work absenteeism.^3^ RPP is often accompanied by systemic symptoms such as nausea, fatigue, and insomnia.^4^ Clinical evidence confirms that PDM is comorbid with affective disorders including anxiety and depression.^5^ A significant positive association was found between dysmenorrhea and depression,^6^ corroborated by a meta-analysis demonstrating that greater dysmenorrhea severity predicts elevated depression and psychological distress.^7^ Together, these observations highlight pain-depression comorbidity in PDM as an important and underrecognized public health concern. This recurrent pelvic pain and its associated affective disturbance constitute a key therapeutic challenge, yet the underlying mechanisms remain poorly understood, partly due to a lack of preclinical models that adequately capture this progression.

Excessive uterine prostaglandin (PG) production, driven by arachidonic acid (AA) metabolism via cyclooxygenase 2 (COX-2) after luteal phase progesterone withdrawal, remains the most widely accepted pathogenesis of PDM.^8^ Non-steroidal anti-inflammatory drugs (NSAIDs) are the first-line therapy, acting as PG synthetase inhibitors, yet up to 15% of patients are non-responders and many experiences adverse effects with long-term use.^9^ Growing evidence suggests that women with PDM exhibit altered pain perception, potentially due to neuroplastic changes driven by cyclic painful episodes.^10^ During menstruation, heightened nociceptor excitability amplifies peripheral pain signals.^11^ Clinically, PDM patients display heightened pain sensitivity and abnormal brain imaging findings, together with higher serum brain-derived neurotrophic factor (BDNF) levels, indicative of nervous system involvement.^12^ Similarly, rodent PDM models show significantly reduced mechanical paw withdrawal thresholds and altered functional brain connectivity.^13^ Maladaptive neuroplasticity may also perturb mood-regulating circuits—via disrupted BDNF and serotonin signaling—thereby contributing to the high comorbidity of depression in PDM patients.^14^ BDNF, abundantly expressed in the female reproductive tract,^15^ modulates pain sensitization through tropomyosin receptor kinase B (TrkB) at spinal and supraspinal levels and is emerging as a key biomarker for chronic pain disorders.^16^

Animal studies provide an essential framework for investigating the mechanisms of chronic pain and informing preclinical therapeutic discovery. Most existing rodent models of pelvic pain rely on a single oxytocin (OT)-induced writhing response, which inadequately reflects the recurrent nature of chronic pelvic pain observed clinically.^17^ To address this limitation, we developed a pharmacologically induced rat model in which cyclic estradiol priming and OT challenge produce a state of RPP. This model is characterized by the cyclically triggered development of hyperalgesia and comorbid depression-like behavior. By applying integrated behavioral, metabolomic, and molecular analyses, we aimed to identify the potential biological alterations underlying the progression from recurrent pain to a persistent comorbid state (graphical abstract).

## Results

### Establishment and characterization of a rat RPP model

**Figure 1:**
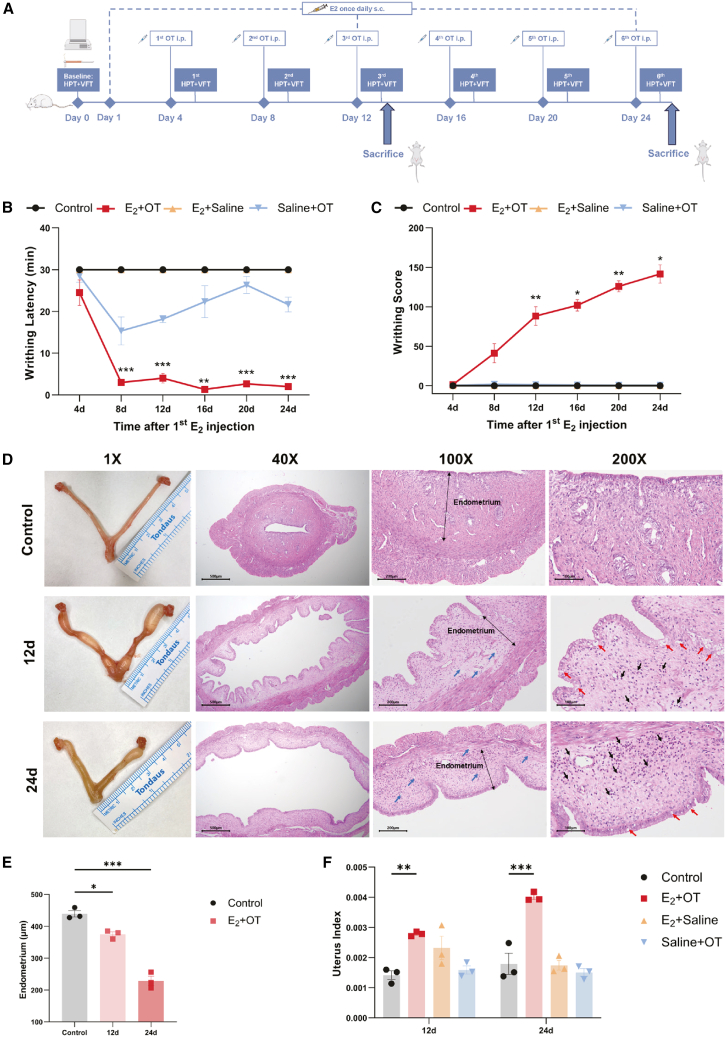
Establishment and characterization of the rat RPP model (A) The flowchart of the RPP rat model induced by estradiol benzoate and oxytocin. HPT, hot plate test; VFT, Von Frey test. (B and C) Writhing latency (B) and writhing score (C) of each rat after oxytocin injection within 30 min (*n* = 3–6). (D) Representative histological images of H&E-stained uterus from the E_2_ + OT group; black arrows indicate inflammatory cell infiltration, red arrows indicate endometrial epithelial damage, and blue arrows indicate edema. Scale bars, 200 μm; all samples were collected during the sustained high-estrogen state that models the late luteal phase in women. (E) Measurement of endometrium thickness in each rat (*n* = 3). (F) Uterus index of each rat (*n* = 3). Data are presented as the mean ± SD; ∗*p* < 0.05, ∗∗*p* < 0.01, ∗∗∗*p* < 0.001, two-way ANOVA (B, C, and F) or one-way ANOVA (E).

To model the recurrent nature of chronic pelvic pain in human PDM, we established a pharmacologically induced rat model of RPP. In this model, sustained estradiol benzoate (E_2_) administration was used to maintain a state of uterine sensitivity. Within this sensitized state, periodic OT induction was administered to trigger repeated pelvic pain, thereby mimicking the cyclic pain condition. Specifically, rats received daily subcutaneous (s.c.) injections of E_2_, with intraperitoneal (i.p.) OT administered 1 h after E_2_ on days 4, 8, 12, 16, 20, and 24 to induce the writhing response (Figure 1A).

The development of RPP episodes was quantified by measuring the writhing response following each OT challenges. As shown in Figures 1B and 1C, on day 8 (cycle 2), the writhing latency in the E_2_ + OT group was significantly shorter than that in the control group (3.00 min vs. 30.00 min, *p* < 0.001), whereas the total writhing scores in the E_2_ + OT group were not significantly different from those in the control group (*p* = 0.056) at this timepoint. Following three cycles (day 12), the E_2_ + OT group exhibited a sustained evident reduction in writhing latency (4 min vs. 30.00 min in the control; *p* < 0.001) and a significant increase in writhing scores (88.30 points vs. 0.00 point in control; *p* = 0.002). The model was further subjected to a total of six cycles (day 24) to provide a more comprehensive assessment of its persistence and phenotypic stability. Notably, the RPP episodes successfully sustained, with the E_2_ + OT group showing significantly elevated writhing scores by day 24 (141.67 points vs. 0.00 point in the control; *p* = 0.020) and a stable, shortened writhing latency across six consecutive cycles.

Next, we performed histological analysis by H&E staining to assess uterine pathology. As shown in Figure 1D, the E_2_ + OT group exhibited progressive and characteristic morphological alterations in the endometrium. After 12 days (three cycles), the uterine structure was moderately disordered, featuring cavity dilation, inflammatory cell infiltration, and endometrial edema. These pathological changes were markedly intensified by day 24 (six cycles), with greater structural disorganization, more pronounced cavity dilation, and sustained inflammatory infiltration. Correspondingly, quantitative analysis confirmed a significant decrease in endometrial thickness (375.2 and 228.7 μm on days 12 and 24, respectively; *p* < 0.001) and a significant increase in the uterine index (0.003 and 0.004 on days 12 and 24, respectively; *p* < 0.001), thereby confirming the establishment of a progressive pathology in the RPP model (Figures 1E and 1F).

Body weight monitoring over the 24-day period revealed that the weight change in the E_2_ + OT group was not significantly altered from that in controls (Figure S1A). To validate the systemic safety of the model, we measured key serum biochemical markers. As shown in Figures S1B and S1C, the levels of alanine aminotransferase (ALT; 46.58 and 52.72 U/L on days 12 and 24, respectively; *p* > 0.05) and aspartate aminotransferase (AST; 155.90 and 158.40 U/L on days 12 and 24, respectively; *p* > 0.05) in the RPP model remained within normal ranges and showed no significant difference from those in the control (ALT: 45.74 U/L; AST: 139.70 U/L). Similarly, no significant alterations were observed in total cholesterol (TC; 1.58 and 1.56 mmol/L on days 12 and 24, respectively; *p* > 0.05) or low-density lipoprotein (LDL; 0.18 and 0.16 mmol/L on days 12 and 24, respectively; *p* > 0.05) levels compared with those in the control (TC: 1.75 mmol/L; LDL: 0.13 mmol/L; Figures S1D and S1E). These results indicate that the consecutive pharmacological regimen used in RPP model did not induce significant hepatotoxicity or disrupt lipid homeostasis.

### PG dysregulation and inflammatory response in the RPP model

**Figure 2:**
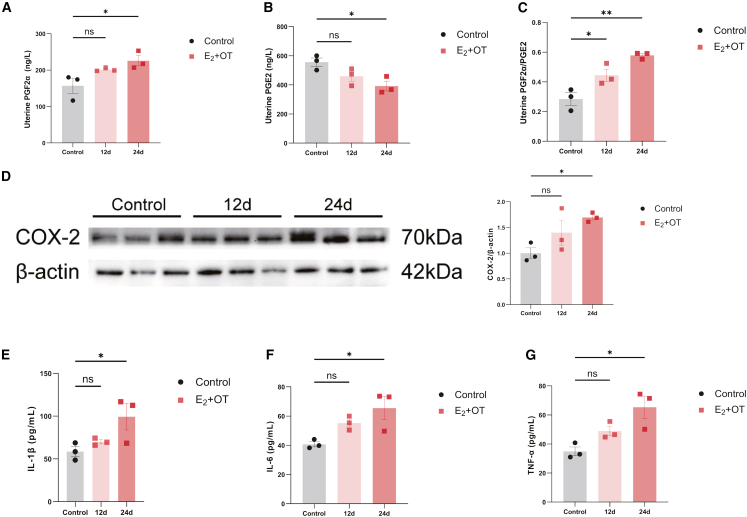
Prostaglandin dysregulation and inflammatory response in the RPP model (A–C) Uterine PGF_2α_ (A), PGE_2_ (B), and PGF_2α_/PGE_2_ ratio (C) on days 12 and 24 of RPP modeling (*n* = 3). (D) The protein levels of COX-2 in the uterine tissue on days 12 and 24 of RPP modeling (*n* = 3). (E–G) The levels of IL-1β (E), IL-6 (F), and TNF-α (G) in the serum on days 12 and 24 of RPP modeling (*n* = 3). Data are presented as the mean ± SD; ∗*p* < 0.05, ∗∗*p* < 0.01, ∗∗∗*p* < 0.001, one-way ANOVA.

**Figure 3:**
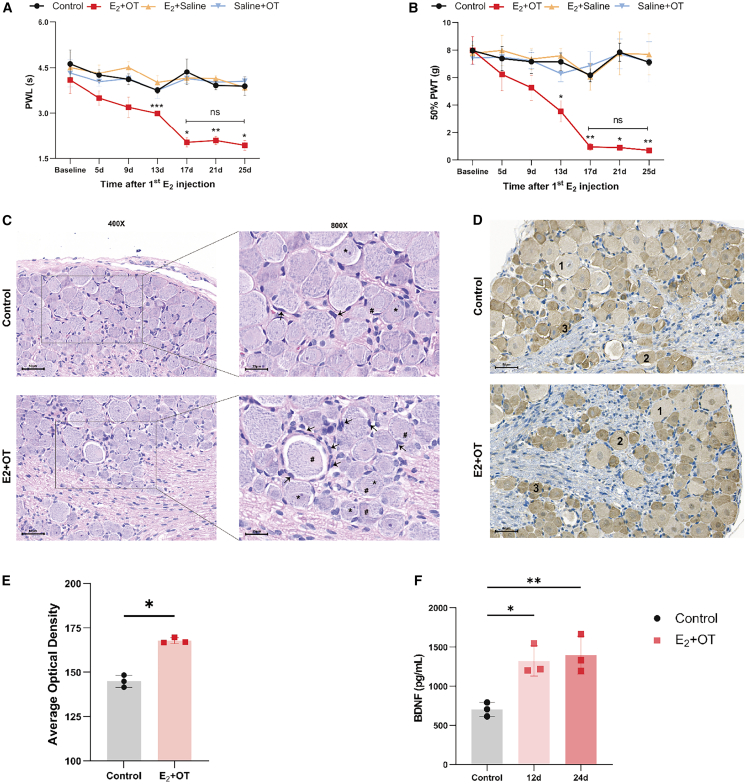
Persistent hyperalgesia in the RPP model (A and B) Paw withdrawal latency (A) and 50% paw withdrawal threshold (B) of each rat (*n* = 3–6). (C) Representative histological images of DRG from T10-S4 by H&E staining. Arrow, Schwann cell; ∗, nuclei; #, cytoplasm. Scale bars, 50 μm and 25 μm. (D and E) Representative IHC images (D) and quantification (E) of BDNF in the DRG from T10-S4. Brown DAB staining indicates BDNF immunopositivity. The staining intensity was semi-quantitatively scored as follows: (1) weak, (2) moderate, and (3) intense. Scale bars, 50 μm. (*n* = 3 biological replicates per group). (F) Serum BDNF level on days 12 and 24 of RPP modeling (*n* = 3). Data are presented as the mean ± SD; ∗*p* < 0.05, ∗∗*p* < 0.01, ∗∗∗*p* < 0.001, two-way ANOVA (A and B) or one-way ANOVA (E and F).

Uterine tissue in the E_2_ + OT group exhibited a progressive imbalance in PG levels compared with the control group, characterized by a significant increase in PGF_2α_ by day 24 (225.80 vs. 157.00 pg/mg, *p* = 0.026) and a decrease in PGE_2_ (391.90 vs. 556.20 pg/mg, *p* = 0.020). This resulted in a significantly elevated PGF_2α_/PGE_2_ ratio on both day 12 (0.44, *p* = 0.030) and day 24 (0.58, *p* = 0.002) compared with those in the control group (0.28), indicating a shift toward a pro-contractile profile (Figures 2A–2C). On day 12, although the individual PGF_2_α and PGE_2_ levels showed a trend of increase and decrease, respectively, these changes were not statistically significant compared with those in the control group (PGF_2α_: 201.40 pg/mg, *p* = 0.121; PGE_2_: 459.20 pg/mg, *p* = 0.134). By day 24, however, the changes in both PGs became pronounced and statistically significant, paralleling the upregulation of COX-2 protein expression in the uterine tissue (Figure 3D).

Additionally, the pro-inflammatory cytokines IL-1β, IL-6, and TNF-α in serum showed a similar temporal pattern: their levels were elevated but not significantly different from those in the control group on day 12 but became significantly elevated by day 24 (IL-1β: 99.28 vs. 58.64 pg/mL, *p* = 0.043; IL-6: 65.49 vs. 40.68 pg/mL, *p* = 0.021; TNF-α: 65.31 vs. 34.92 pg/mL, *p* = 0.010), indicating the development of a systemic inflammatory response in the RPP model over time (Figures 2E–2G).

### Persistent hyperalgesia in the RPP model

To quantitatively assess the chronic pain state in the RPP model, we measured mechanical and thermal nociceptive thresholds by using the Von Frey test (VFT) and hot plate test (HPT), respectively. After three cycles (day 13), compared with the control (Figures 3A and 3B), the E_2_ + OT group showed significantly reduced mechanical paw withdrawal threshold (PWT) (3.54 g vs. control 7.14 g, *p* = 0.015) and thermal paw withdrawal latency (PWL) (2.99 s vs. 3.75 s in the control, *p* < 0.001). Intriguingly, the tendency of decreased pain thresholds remained stable through six cycles by day 25 (PWT: 0.70 g vs. control 7.11 g, *p* < 0.001; PWL: 1.93 s vs. 3.88 s in the control, *p* = 0.023), without statistical differences in the PWL and PWT observed between the last three cycles, indicating the establishment of persistent hyperalgesia in the RPP model.

The dorsal root ganglion (DRG)—a primary site of peripheral nociceptive signaling and BDNF synthesis—showed histopathological alterations in RPP rats (Figure 3C). Compared with controls, DRG in the E_2_ + OT group exhibited Schwann cell proliferation around neuronal soma, eccentric nuclei and enlargement, and lacy appearance of cytoplasm, indicating neuronal degeneration. Immunohistochemical (IHC) analysis demonstrated a significantly higher density of BDNF-positive neurons in the DRG of the E_2_ + OT group than in the control (Figures 3D and 3E). These histopathological features are characteristics of chronic pain models and indicative of peripheral neuronal sensitization underlying hyperalgesia. Most BDNF-positive neurons in the control group were characterized by weak to moderate staining, whereas intense staining of BDNF was detected in the E_2_ + OT group. The observed BDNF overexpression within the DRG, a key hub for pain processing, suggests its potential contribution to the amplification of nociceptive signaling in the RPP model. As shown in Figure 3F, serum BDNF levels were significantly elevated in the RPP model on day 12 (1321.34 pg/mL, *p* = 0.012) and day 24 (1396.27 pg/mL, *p* = 0.007) compared with the control (703.61 pg/mL), indicating a systemic correlate of enhanced pain sensitization in the RPP model. Collectively, these results demonstrate that six cycles of writhing responses over 24 days pharmacologically induce stable hyperalgesia, accompanied by neurochemical and pathological changes, in the peripheral nervous system.

### Comorbid depression-like behavior in the RPP model

An independent cohort with increased sample size (*n* = 8 per group) confirmed the reproducibility of the RPP model, exhibiting consistent writhing responses, uterine PG dysregulation, and persistent hyperalgesia (Figure S2).

**Figure 4:**
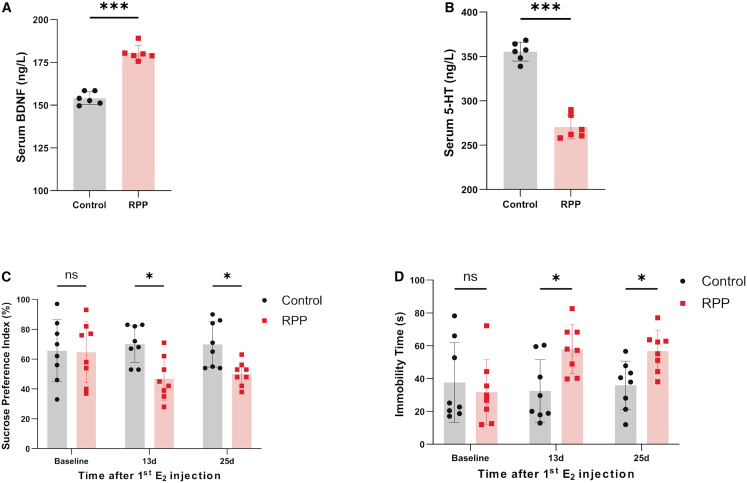
Comorbid depression-like behavior in the RPP model (A and B) Serum BDNF (A) and 5-HT (B) levels in the RPP model (*n* = 6). (C and D) Sucrose preference ratio (C) and immobility time (D) in the RPP model (*n* = 8). Data are presented as the mean ± SD; ∗*p* < 0.05, ∗∗*p* < 0.01, ∗∗∗*p* < 0.001, paired *t* test (A and B) or two-way ANOVA (C and D).

Given BDNF’s role in neuroinflammation and mood regulation (Figure 4A), we next assessed affective behavior and serum serotonin (5-HT). RPP rats displayed significantly reduced serum 5-HT levels (270.28 vs. 255.34 ng/mL in the control, *p* < 0.001; Figure 4B). Concomitantly, sucrose preference test (SPT) revealed a significant decrease in sucrose preference on both day 13 (46.88% vs. 70.13% in the control, *p* = 0.012) and day 25 (50.13% vs. 69.88% in the control, *p* = 0.024), indicating the existence of anhedonia in the RPP model (Figure 4C). Furthermore, the RPP group showed a significant increase in immobility time in the forced swim test (FST) both on day 13 (57.9 s vs. 32.5 s in the control, *p* = 0.032) and day 25 (56.8 s vs. 35.9 s in the control, *p* = 0.025), demonstrating behavioral despair in the RPP model (Figure 4D). Taken together, these data confirm the development of comorbid depression-like behavior in the RPP model, paralleling persistent hyperalgesia.

### Serum metabolomics in the RPP model

**Figure 5:**
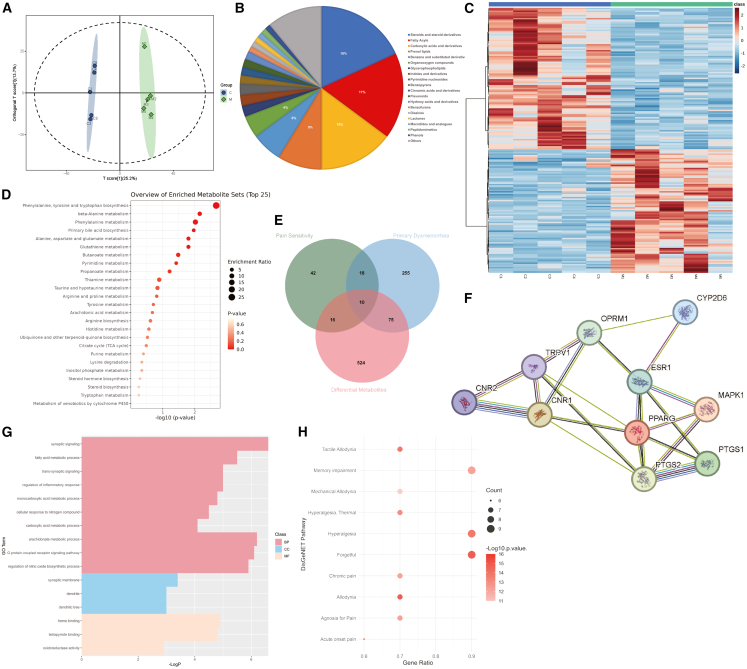
Serum metabolomics in the RPP model (A) OPLS-DA analysis of serum metabolomics in the control and RPP groups (*n* = 5). (B) Ontology of the identified differential metabolites based on the ClassyFire website. (C) Heatmap of significant differential metabolites. (D) KEGG enrichment analysis of differential metabolites. (E) Venn diagram of the overlapping targets of “primary dysmenorrhea,” “pain sensitivity,” and “differential metabolites.” (F) PPIs of the overlapping targets via the STRING database. (G) GO enrichment analysis of the overlapping targets in biological process (BP), cellular component (CC), and molecular function (MF). (H) DisGeNET enrichment analysis of the overlapping targets.

To explore systemic metabolic alterations, we conducted untargeted serum metabolomics by UPLC-Q-Orbitrap-MS. Orthogonal Partial Least Squares Discriminant Analysis (OPLS-DA) analysis revealed notable differences in serum metabolism between the control and RPP groups (Figure 5A). By applying a significance level of *p* < 0.05 and screening criteria of fold change (FC) > 2 or <0.5, a total of 234 metabolites were identified, with differential metabolites predominantly classified into steroids and steroid derivatives, fatty acyls, and carboxylic acids/derivatives (Figures 5B and 5C). KEGG enrichment analysis uncovered significant metabolic reprogramming in the RPP model (Figure 5D), highlighting amino acid metabolism pathways—including phenylalanine, tyrosine, and tryptophan biosynthesis, as well as arginine and proline metabolism. These pathways contribute to neurotransmitter synthesis (e.g., 5-HT and dopamine) and were also linked to AA metabolism and steroid biosynthesis.

Furthermore, we integrated known targets for PDM and pain sensitivity (GeneCards) and predicted targets of differential metabolites (ChEMBL). As illustrated in Figures 5E and 5F, 10 overlapping targets were identified, which were ESR1, PTGS1, PTGS2, MAPK1, TRPV1, PPARG, CNR1, CNR2, OPRM1, and CYP2D6. GO functional enrichment analysis revealed that these targets are primarily involved in fatty acid metabolic processes, regulation of inflammatory responses, and nitric oxide biosynthesis, which are hallmarks of PDM (Figure 5G). Notably, these targets also participate in synaptic signaling and localize to the synaptic membrane and dendrites. DisGeNET pathway analysis further linked these targets to chronic pain, acute-onset pain, hyperalgesia, and allodynia (Figure 5H), which represent key characteristics of pain hypersensitivity that correlates with BDNF upregulation in the RPP model. In a word, the metabolomic profile further supports progressive nociceptive sensitization in the RPP model. Moreover, it reveals alterations in neurotransmitter biosynthesis pathways underlying pain-depression comorbidity, highlighting key targets and pathways for future investigation.

## Discussion

The aim of this study was to establish and characterize a pharmacologically induced rat model of RPP. Most published rodent models have relied on short-term estrogen priming and a single OT induction to elicit transient pain, failing to capture the recurrent nature of chronic pelvic pain.^17^ Recent attempts to develop chronic models, such as the protocol employing consecutive high-dose E_2_ injections, have been limited by declining writhing responses over subsequent cycles, indicating a lack of stable phenotypic persistence.^18^ Similarly, other approaches involving extended estradiol dosing or varied OT schedules have not succeeded in producing consistent long-term pain.^13^ The absence of a reproducible animal model that integrates cyclic pain induction with the development of long-term affective comorbidity has hindered the investigation of the underlying pathophysiology and therapeutic discovery.

To address this, we established a pharmacological induced RPP model by using cyclic E_2_ and OT administration over a 24-day period for six cycles. The specific dosing regimen (daily E_2_ priming with 1.25 mg/kg, elevated to 2.5 mg/kg co-administered with 10 U/kg OT on challenge days) was optimized based on established protocols in the literature to induce sustained uterine sensitization and recurrent pain episodes.^13^^,^^17^^,^^18^^,^^19^ This protocol enabled repeated induction of pelvic pain, which stabilized from the third cycle (day 12) onward, achieving a model that captures the cyclicity of chronic pain (Figure 1). The day 12 timepoint was selected as it represents the establishment of a RPP phenotype, while day 24 demonstrates the persistent hyperalgesia and comorbid depression-like behavior.

Furthermore, this RPP model recapitulated key features of a chronic pain state with heightened pain sensitivity characteristic. It is important to notice that in healthy individuals, normal uterine contractions during menstruation remain below pain perception thresholds and are not perceived as clinically painful. In contrast, PDM arises from PG-mediated uterine hypercontractility and ischemia, and it sensitizes peripheral nociceptors. This peripheral sensitization induces hyperalgesia, resulting in clinically significant menstrual pain. In our study, RPP rats exhibited progressive hyperalgesia (Figures 3A and 3B), showing significant reductions in both thermal and mechanical pain thresholds by cycle 3 (day 12), with this downward trend persisting throughout the six cycles (day 24). BDNF,^20^ a member of the neurotrophin family, plays a critical role in pain sensitization across multiple nociceptive pathways at both spinal and supraspinal levels.^21^ Upon release from DRG, BDNF binds to TrkB located on primary afferent nerve terminals and postsynaptic neurons in the spinal cord.^22^ In our model, this was supported by elevated serum BDNF levels and histological observations of microstructural alterations alongside increased BDNF-positive neurons in the DRG (Figure 4). The observed BDNF overexpression in the DRG suggests a potential role in peripheral sensitization that possibly amplifies menstrual pain in PDM. Beyond the persistent hyperalgesia, RPP rats exhibited affective changes resembling depression, reflecting the well-documented comorbidity between chronic pain and affective disorders.^23^ Behavioral tests showed a significant decrease in sucrose preference (anhedonia) and a prolonged immobility time in FST (behavioral despair), which were coupled with a concurrent reduction in serum 5-HT levels (Figure 5). These results mirror the frequent clinical co-occurrence of chronic pain and affective disorders^5^ and, within our model, point to a potential interplay between peripheral sensitization and systemic neurochemical dysregulation.

Nevertheless, the interpretation of these systemic biomarkers requires caution, particularly in light of their complex and compartment-specific roles. This is especially true for BDNF, which exhibits distinct functions in peripheral and central nervous systems.^24^ While its upregulation in the DRG is linked to pro-nociceptive effects^25^ and facilitates pain transmission,^26^ its role in the brain, particularly in regions like the hippocampus and prefrontal cortex, is often associated with neuroplasticity and anti-depressant effects.^27^ Therefore, the co-existence of elevated serum BDNF—which may primarily reflect peripheral release—with depression-like behavior in the RPP model underscores the spatial and functional complexity of BDNF signaling. This apparent paradox aligns with clinical observations of its dual role in chronic pain-depression comorbidity, where peripheral sensitization and central affective dysregulation may involve a divergent BDNF pathway.^28^ Similarly, this is the case for the interpretation of systemic serotonin. We acknowledge that changes in serum 5-HT concentrations do not directly or causally reflect central serotonergic function or depressive behavior.^29^^,^^30^ Accordingly, the observed reduction is best interpreted as a correlative indicator of systemic serotonergic dysregulation, potentially linked to the metabolomic perturbations in its precursor, tryptophan, rather than as direct mechanistic evidence of central mood dysregulation.^31^ To fully elucidate the distinct contributions of these signaling molecules to the peripheral and central dimensions of chronic pelvic pain, future studies incorporating region-specific quantification of BDNF and serotonin within key brain regions (e.g., hippocampus and prefrontal cortex) will be essential. Finally, it is important to note that while peripheral OT administration in our RPP model primarily targets uterine contractions, its potential direct or indirect central effects on affective and pain circuits cannot be entirely ruled out and need further study.^32^

The potential link between peripheral pathology and central nervous system modulation in RPP model is further supported by the untargeted metabolomic profile (Figure 5). The enrichment of phenylalanine, tyrosine, and tryptophan biosynthesis pathways suggests a perturbation in the pool of monoamine neurotransmitter precursors, providing a plausible metabolic basis for the observed serotonin reduction and depression-like behavior. Additionally, the dysregulation of AA and steroid metabolism pathways aligns with and potentially sustains the inflammatory milieu suggested by our other findings (Figure 2). Notably, the integration of metabolite-target networks pinpointed key molecules (e.g., CNR1/2 and OPRM1) enriched in synaptic signaling and pain pathways, functionally linking peripheral metabolic shifts to pain sensitization mechanisms in the RPP model. These findings point to a multifaceted interplay involving peripheral metabolic reprogramming that may concurrently relate to inflammatory processes and disrupt neurotransmitter homeostasis, thereby contributing to the persistence of both pain and affective symptoms in the RPP model. Thus, this model may serve as a valuable platform for evaluating dual-target medical interventions aimed at both pain perception and affective disorders.

In this study, we established and characterized a pharmacologically induced rat model of RPP that captures key features of a chronic, cyclic pain condition, including persistent hyperalgesia and comorbid depression-like behavior. Our findings implicate BDNF overexpression in the DRG and serum, as well as concomitant reduced serotonin as potential mechanisms underlying this pain-depression comorbidity. As such, this RPP model provides a valuable translational platform for investigating the mechanisms and for screening therapeutic targets for combined pain and affective symptoms in clinical practice.

### Limitations of the study

Evidently, this study has several important limitations that define the scope and future direction of this work. First and foremost, the RPP model is pharmacologically induced and does not replicate the natural physiological or disease state of PDM. While it captures features of recurrent pain and affective comorbidity, its construct validity is based on hormonal and contractile challenges, rather than the complete pathophysiology of any specific human disorder. Second, the dosing regimen of E_2_ and OT was optimized based on established protocols in the literature to induce sustained uterine sensitization and recurrent pain, rather than from a comprehensive pharmacodynamic analysis, which represents a focus for future investigation. Third, our findings are primarily correlative and descriptive; the study lacks interventional experiments to definitively establish causal mechanisms underlying the observed pain and comorbid depression-like behaviors. Fourth, this study mainly focused on neurotrophic pathways and peripheral nervous system, but other inflammatory or neurotransmitter pathways likely contribute to the pain-depression interaction. Meanwhile, long-term peripheral nociceptive input may lead to changes in central plasticity, and the mechanisms involving the central nervous system in the RPP model need further exploration. These limitations precisely highlight the necessity for future research to validate the translational relevance of findings from this model and to deepen the mechanistic understanding of chronic pain disorders.

## Resource availability

### Lead contact

Requests for further information and resources should be directed to and will be fulfilled by the lead contact, Huifang Zhou (zhouhuifang2011301@163.com).

### Materials availability

This study did not generate new unique reagents.

### Data and code availability

• Metabolomics data have been deposited at MetaboLights and are publicly available as of the date of publication. Accession number is listed in the key resources table. All data reported in this paper are available from the lead contact upon request.
• This paper does not report original code.
• Any additional information required to reanalyze the data reported in this paper is available from the lead contact upon request.

## Acknowledgments

This work was supported by the 10.13039/501100001809National Natural Science Foundation of China (nos. 82205167 and 82474567).

## Author contributions

Conceptualization, H.F. Zhou and B.Liu; methodology, X.T. Yang and Y.J. Qin; investigation, X.T. Yang, Y.J. Qin, Q.Zhao, and H.J. Zhao; writing – original draft, X.T. Yang and Y.J. Qin.; writing – review & editing, H.F. Zhou and B.Liu; funding acquisition, H.F. Zhou and B.Liu; resources, H.J. Zhao and Y.Y. Ding.; supervision, Y. Y. Ding, H.F. Zhou, and B.Liu.

## Declaration of interests

The authors declare no competing interests.

## Declaration of generative AI and AI-assisted technologies in the writing process

During the preparation of this work, the author(s) used DeepSeek AI Assistant in order to improve language and readability of this article. After using this tool, the author(s) reviewed and edited the content as needed and take full responsibility for the content of the publication.

## STAR★Methods

### Key resources table

**Table:** 

REAGENT or RESOURCE	SOURCE	IDENTIFIER
**Antibodies**		

Anti-COX2 antibody	Cell Signaling Technology	Cat# 12282; RRID: AB_2164118
Anti-BDNF antibody	Abcam	Cat# ab108319; RRID: AB_10862052
Beta Actin Monoclonal antibody	Proteintech	Cat# 66009-1-Ig; RRID: AB_2687938
HRP-conjugated Goat Anti-Rabbit IgG	Proteintech	Cat# SA00001-2; RRID: AB_2722564

**Chemicals, peptides, and recombinant proteins**		

Estradiol Benzoate Injection	NSHF	Cat# 2111272
Oxytocin Injection	BBCA Pharmaceutical	Cat# 210819-2

**Critical commercial assays**		

Rat PGF_2_α ELISA Kit	MEIMIAN	Cat# MM-0230R1
Rat PGE_2_ ELISA Kit	MEIMIAN	Cat# MM-0068R1
Rat BDNF ELISA Kit	Elabscience	Cat# E-EL-R1235
Rat IL-6 ELISA Kit	Elabscience	Cat# E-EL-R0015
Rat IL-1β ELISA Kit	Elabscience	Cat# E-EL-R0012
Rat TNF-α ELISA Kit	Elabscience	Cat# E-EL-R2856
Rat 5-HT ELISA Kit	Afantibody	Cat# AF2443-A
BCA Protein Assay Kit	Yeasen Biotechnology	Cat# 20201ES76

**Deposited data**		

Metabolomics raw data	Metabolights	[Metabolights]: MTBLS13869

**Experimental models: Organisms/strains**		

Sprague-Dawley rat, female	Beijing Vital River Laboratory Animal Technology Co., Ltd.	Production License: SCXK (ZHE) 2019-0001

**Software and algorithms**		

GraphPad Prism	GraphPad Software	Version 10.1.2; RRID:SCR_002798
R	The R Foundation	Version 4.0.3; RRID:SCR_001905
ImageJ	NIH	RRID:SCR_003070
MS-DIAL	N/A	https://prime.psc.riken.jp/compms/msdial/main.html
STRING database	N/A	Version 11.5; RRID:SCR_005223
Metascape	N/A	RRID:SCR_016620
jvenn	N/A	https://www.bioinformatics.com.cn/static/others/jvenn

### Experimental model and study participant details

A total of forty (n = 40) specific pathogen-free (SPF) female Sprague-Dawley rats (5 weeks old, 150 ± 10 g) were used. Animals were purchased from Beijing Vital River Laboratory Animal Technology Co., Ltd. (Production License: SCXK (ZHE) 2019-0001). Rats were housed in a controlled barrier environment at the Experimental An-imal Center of Nanjing University of Chinese Medicine with a temperature of 19-25°C, relative humidity of 40%-70%, and a 12/12 hr light/dark cycle. Food and water were provided *ad libitum*. All procedures were approved by the Animal Ethics Committee of Nanjing University of Chinese Medicine (Ethical Approval Number: ACU211205) and were conducted in accordance with institutional guidelines.

### Method details

#### Establishment of recurrent pelvic pain (RPP) rat model

Rats with regular estrous cycles, as determined by daily vaginal smear cytology, were selected for experiments. In Experiment 1, 24 rats were randomly assigned to four groups (n=6 per group): Control (Saline s.c. + Saline i.p.), E_2_ + OT (RPP model group), E_2_ + Saline, and Saline + OT. In Experiment 2, 16 rats were divided into Control and RPP groups (n=8 per group). The sample size for each experiment and measurement is explicitly stated in the corresponding figure legend.

The RPP model was induced via subcutaneous (s.c.) injections of estradiol benzoate (E_2_) and intraperitoneal (i.p.) injections of oxytocin (OT). To maintain a state of persistent uterine sensitization, all rats in the E_2_-treated groups received once-daily s.c. injections of E_2_. The dosing regimen was designed to provide a higher sensitizing dose on days coinciding with the OT challenge: A dose of 2.5 mg/kg was administered on days 1, 4, 8, 12, 16, 20, and 24. On all other days, a maintenance dose of 1.25 mg/kg was used. Control rats received an equivalent volume of saline s.c. daily.

To simulate recurrent pelvic pain episodes, rats received i.p. injections of OT (10 U/kg) or saline on days 4, 8, 12, 16, 20, and 24. Each OT or saline injection was administered 1 hour after the E_2_ or saline injection on those specific days. The writhing response was observed and recorded immediately following the OT challenge.

#### Sample collection

On day 12, 24, or 25 (post-behavioral tests), rats were anesthetized with 3% pentobarbital sodium (45 mg/kg, i.p.). All sample collections were performed 24 hours after the final E_2_ and OT co-administration. At this time point, the exogenous hormone regimen consistently maintained the rats in a state of high estrogen, which is designed to mimic the hormonal environment of the late luteal phase in women. Blood was collected from the abdominal aorta, and serum was separated by centrifugation. Uterine tissues and lumbosacral dorsal root ganglia (DRG, T10-S4) were rapidly dissected, flash-frozen in liquid nitrogen, and stored at -80°C for subsequent analysis.

#### Behavioral tests

All behavioral tests were performed by investigators blinded to the group assignments.

##### Writhing response

Following each OT injection (on days 4, 8, 12, 16, 20, and 24), writhing episodes were recorded for 30 min. Latency (time to the first writhe) and a weighted score (Level 1 x1, Level 2 x2, Level 3 x3) were calculated.

##### Hot plate test (HPT)

Thermal nociception was assessed by recording the paw withdrawal latency (PWL) to a thermal stimulus (54.0 ± 0.1°C). Tests were performed on days 5, 9, 17, 21, and 25.^33^

##### Von frey test (VFT)

Mechanical nociception was assessed by determining the 50% mechanical paw withdrawal threshold (PWT) using the up-down method.^34^ Tests were performed on days 5, 9, 17, 21, and 25.

##### Forced swim test (FST)

Depression-like behavior was assessed on days 13 and 25. Rats were placed individually in a water-filled cylinder (23-25°C), and the total immobility time during the 5-min test period was recorded.^35^

##### Sucrose preference test (SPT)

Anhedonia, a core symptom of depression, was evaluated on days 13 and 25. After acclimation and water deprivation, rats were presented with two bottles containing 1% sucrose solution and water, respectively. Sucrose preference was calculated as [sucrose intake / (sucrose intake + water intake)] × 100% over a 12-hr test period.^36^

#### Histology (H&E staining)

Uterine and DRG tissues were fixed in 4% paraformaldehyde, paraffin-embedded, and sectioned at 5 μm. Sections were deparaffinized, stained with Hematoxylin and Eosin (H&E) using standard protocols, and imaged under a light microscope.

#### Enzyme-linked immunosorbent assay (ELISA)

Serum levels of PGF_2_α, PGE_2_, BDNF, IL-6, IL-1β, TNF-α, and 5-HT were quantified using commercial ELISA kits according to the manufacturers' instructions. Absorbance was measured using a microplate reader.

#### Western blotting

Tissue proteins were extracted, quantified by BCA assay, separated by SDS-PAGE, and transferred to PVDF membranes. Membranes were blocked and incubated overnight at 4°C with primary antibody against COX-2 (1:1000). After incubation with an HRP-conjugated secondary antibody (1:10,000), protein bands were visualized using chemiluminescence.

#### Immunohistochemistry (IHC)

Deparaffinized and rehydrated uterine/DRG sections underwent antigen retrieval, peroxidase quenching, and blocking. Sections were incubated with anti-BDNF antibody (1:500) overnight at 4°C, followed by a secondary antibody. BDNF expression was visualized with DAB, counterstained with hematoxylin, and imaged. The average optical density (AOD) was quantified from six random fields per sample using ImageJ software.

#### Untargeted serum metabolomics

Serum metabolites were extracted with cold methanol: acetonitrile (1:1, v/v). LC-MS analysis was performed on a Shimadzu Nexera X2 UHPLC system coupled to a Thermo Q Exactive Plus mass spectrometer equipped with a HSS T3 column (2.1 × 100 mm, 1.8 μm). Data were acquired in both positive and negative ionization modes. Raw data were processed using MS-DIAL for peak picking, alignment, and annotation against HMDB and MassBank databases. Multivariate statistical analysis (OPLS-DA) was performed on Pareto-scaled data.

#### Target collection and enrichment analysis

Metabolite-associated targets were retrieved from ChEMBL. PDM- and pain sensitivity-related targets were collected from GeneCards. Overlapping targets were analyzed for protein-protein interactions (PPI) using the STRING database. Functional enrichment analysis (GO, KEGG, DisGeNET) was performed using Metascape and visualized in R.

### Quantification and statistical analysis

Data are presented as mean ± standard deviation (SD). Statistical analysis was per-formed using GraphPad Prism (v10.1.2) and R (v4.0.3). Differences between two groups were analyzed by unpaired Student's t-test. Comparisons among multiple groups were analyzed by one-way ANOVA followed by Dunnett's or Tukey's post hoc test. Time-course data were analyzed by two-way repeated measures ANOVA with Bonferroni's post hoc test. A *p*-value < 0.05 was considered statistically significant. The specific statistical test used for each experiment, along with the exact n value (number of biological replicates), is detailed in the corresponding figure legend.
