# Supplementary material for: A pharmacological rat model of recurrent pelvic pain exhibiting hyperalgesia and depression-like behaviors
Source: iScience. 2026 Mar 5;29(4):115059. doi: 10.1016/j.isci.2026.115059 (PMC13015246; doi:10.1016/j.isci.2026.115059)

iScience, Volume *29*

Supplemental information

A pharmacological rat model of recurrent pelvic pain exhibiting hyperalgesia and depression-like behaviors

Xiaotian Yang (杨晓田), Yajie Qin (秦雅洁), Qi Zhao (赵琪), Huiijin Zhao (赵绘锦), Yinyin Ding (丁银银), Bei Liu (刘贝), and Huifang Zhou (周惠芳)

Figure S1: Systemic safety of the RPP Model.

(A) Weight change during modeling (n = 3 ~ 6); (D-G) Serum ALT, AST, TC, LDL levels of in the 12-days and 24-days of RPP modeling (n = 3). Data are presented as mean ± SD, *p< 0.05, ** p < 0.01, *** p < 0.001 by two-way ANOVA (A) or by one-way ANOVA (B, C, D and E).

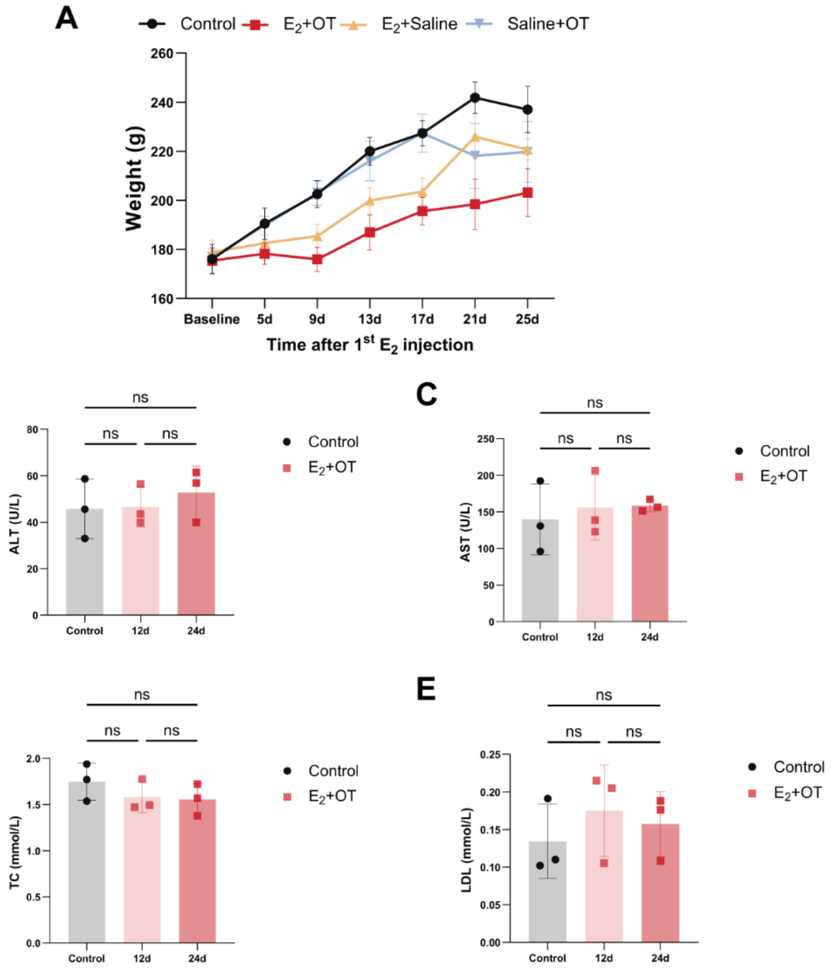

Figure S2: Validation of the RPP Model.

(A) Weight change during modeling (n = 8); (B and C) Writhing Latency (B) and writhing score (C) of each rat after oxytocin injection within 30 minutes (n=8); (D and E) Paw withdrawal latency (D) and 50% paw withdrawal threshold (E) of each rat (n=6); (F, G and H) Uterine $PGF_{2\alpha}$ (F), PGE_2 (G) and $PGF_{2\alpha}/PGE_2$ (H) of the RPP model (n=6); (I) Representative histological images by H&E staining, Scale bar = 200 μm. Data are presented as mean ± SD, * $p < 0.05$, ** $p < 0.01$, *** $p < 0.001$ by two-way ANOVA (A, B, C, D and E) or by one-way ANOVA (F, G and H).

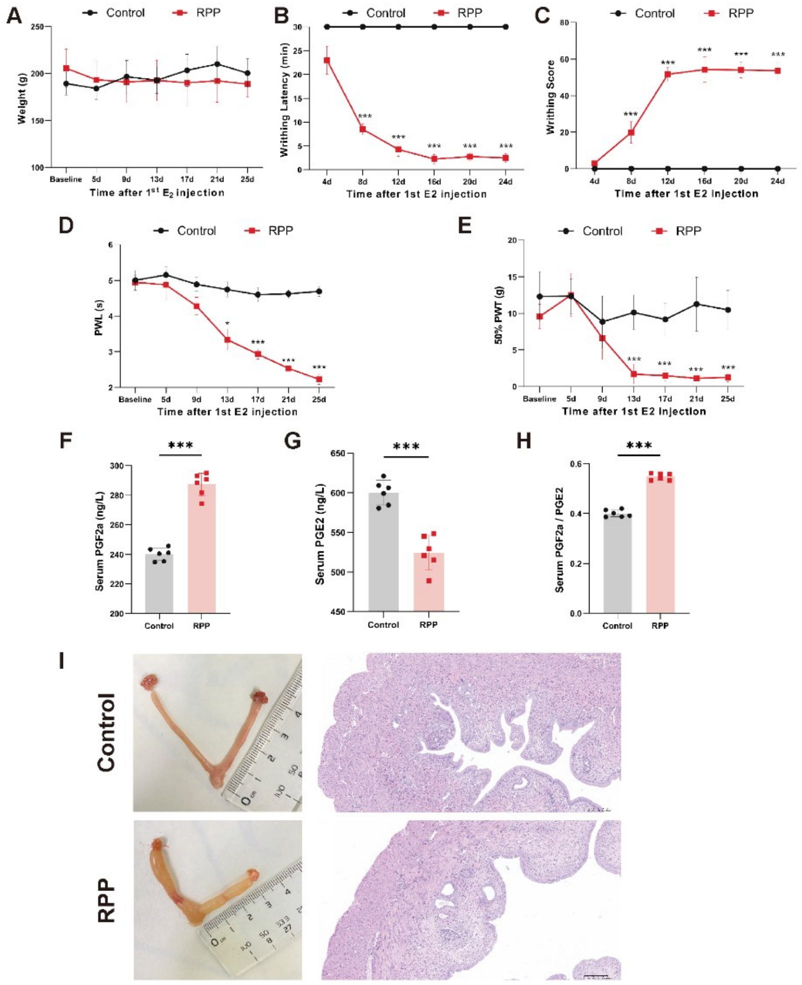

Supplement: Document S1. Figures S1 and S2 [file mmc1.pdf]
